# EmTIP, a T-Cell Immunomodulatory Protein Secreted by the Tapeworm *Echinococcus multilocularis* Is Important for Early Metacestode Development

**DOI:** 10.1371/journal.pntd.0002632

**Published:** 2014-01-02

**Authors:** Justin Komguep Nono, Manfred B. Lutz, Klaus Brehm

**Affiliations:** 1 University of Würzburg, Institute for Hygiene and Microbiology, Würzburg, Germany; 2 University of Würzburg, Institute of Virology and Immunobiology, Würzburg, Germany; McGill University, Canada

## Abstract

**Background:**

Alveolar echinococcosis (AE), caused by the metacestode of the tapeworm *Echinococcus multilocularis*, is a lethal zoonosis associated with host immunomodulation. T helper cells are instrumental to control the disease in the host. Whereas Th1 cells can restrict parasite proliferation, Th2 immune responses are associated with parasite proliferation. Although the early phase of host colonization by *E. multilocularis* is dominated by a potentially parasitocidal Th1 immune response, the molecular basis of this response is unknown.

**Principal Findings:**

We describe EmTIP, an *E. multilocularis* homologue of the human T-cell immunomodulatory protein, TIP. By immunohistochemistry we show EmTIP localization to the intercellular space within parasite larvae. Immunoprecipitation and Western blot experiments revealed the presence of EmTIP in the excretory/secretory (E/S) products of parasite primary cell cultures, representing the early developing metacestode, but not in those of mature metacestode vesicles. Using an *in vitro* T-cell stimulation assay, we found that primary cell E/S products promoted interferon (IFN)-γ release by murine CD4+ T-cells, whereas metacestode E/S products did not. IFN-γ release by T-cells exposed to parasite products was abrogated by an anti-EmTIP antibody. When recombinantly expressed, EmTIP promoted IFN-γ release by CD4+ T-cells *in vitro*. After incubation with anti-EmTIP antibody, primary cells showed an impaired ability to proliferate and to form metacestode vesicles *in vitro*.

**Conclusions:**

We provide for the first time a possible explanation for the early Th1 response observed during *E. multilocularis* infections. Our data indicate that parasite primary cells release a T-cell immunomodulatory protein, EmTIP, capable of promoting IFN-γ release by CD4+ T-cells, which is probably driving or supporting the onset of the early Th1 response during AE. The impairment of primary cell proliferation and the inhibition of metacestode vesicle formation by anti-EmTIP antibodies suggest that this factor fulfills an important role in early *E. multilocularis* development within the intermediate host.

## Author Summary

*E. multilocularis* is a parasitic helminth causing the chronic human disease alveolar echinococcosis. Current disease control measures are very limited resulting in a high case-fatality rate. A transiently dominating Th1 immune response is mounted at the early phase of the infection, potentially limiting parasite proliferation and disease progression. Understanding the molecular basis of this early anti-*Echinococcocus* Th1 response would provide valuable information to improve disease control. The authors found that EmTIP, a T-cell immunomodulatory protein homologue, is secreted by the parasite early larva and promotes a Th1 response in host cells. Interestingly, EmTIP binding by antibodies impairs the development of the early parasite larva towards the chronic stage. Altogether the authors propose that *E. multilocularis* utilizes EmTIP for early larval development, but in the process, the factor is released by the parasite larva and influences host T-cells by directing a parasitocidal Th1 immune response. Therefore, the authors recommend EmTIP as a promising lead for future studies on the development of anti-*Echinococcus* intervention strategies.

## Introduction

Alveolar echinococcosis (AE), resulting from the formation, establishment and dissemination of the metacestode (MV) larval stage of the fox tapeworm *E. multilocularis* is considered one of the most severe human parasitoses in the world [1], [2]. Upon oral ingestion of parasite-derived, infective eggs by intermediate hosts (rodents and, occasionally, humans), the oncosphere larva is activated, hatches, and penetrates the intestinal barrier, usually evoking a Th1-dominated immune response with IFN-γ associated immune effector functions [3]. Within the liver of the intermediate host, the oncosphere then undergoes a metamorphosis toward the bladder-like metacestode larval stage which grows infiltratively, like a malignant tumor, into the surrounding host tissue. During this process, the early Th1 response is gradually replaced by a Th2 response, dominated by interleukin (IL)-5 and IL-10 [4]. AE has a high case-fatality rate and is associated with severe morbidity. The implementation of benzimidazole-based chemotherapy has markedly improved the prognosis of patients [1], [2]. However, this treatment only proved to be parasitostatic [5]–[7], requiring long-term to life-long administration [8]. Currently, AE therapy is modestly satisfactory [1], [2]. Alternative targets for therapy are thus desperately needed.

A defining feature of the disease is the modulation of the host immune response by the parasite larvae as reflected by its widely accepted polar character [4], [9], [10]. Current hypotheses are that a Th1 response is parasitocidal, whereas a Th2 response associates with parasite growth and disease progression [4], [9], [10]. This general picture is supported by previous studies which compellingly showed that the resistance of murine [11]–[13] or human [14], [15] hosts to *E. multilocularis* metacestodes is associated with a Th1-dominated immune response whereas a Th2-dominated immune response occurs as *E. multilocularis* metacestodes thrive in these murine [16], [17] or human [15], [18], [19] hosts. In agreement with a parasitocidal role for Th1 responses during AE, administration of Th1-inducing immune-stimulants like Bacillus Calmette–Guérin (BCG) [20]–[23], IL-12 [13], IFN-α-2a [11], [24] and IFN-γ [25], [26] have all been shown to restrain parasite establishment, proliferation or dissemination in rodents experimentally infected with *E. multilocularis* larvae. Conversely, Th2-dominated immune responses have been tightly associated with progressive forms of AE both in humans [15], [19] and mice [16], [17] with compelling evidence pointing at an underlying expansion of regulatory T-cells [27], [28]. As a result of these investigations, the current picture of a parasitocidal Th1 response and a permissive Th2 host response during AE has gradually emerged [4], [9], [29], [30]. This picture has proven to be largely representative of the immunity to AE when considering studies which report the reversal of the dominating Th2 response to a Th1 response in patients with spontaneously died out lesions [14], [15], [31]. Understanding the basis of the anti-parasitic Th1 immune response would provide a wealth of information to interfere with AE. Although, like *E. multilocularis*, several tissue-dwelling parasitic helminths have been reported to show a similar Th1/Th2 immune polarity [32]–[34], the molecular basis for the early onset of the host protective Th1 response remains unknown.

We have developed an *in vitro* system for the cultivation of *E. multilocularis* primary cells that closely mimics the oncosphere-metacestode transition as it occurs early during an infection [35], [36]. In the primary cell cultivation system, metacestode vesicles are formed from parasite stem cells within 1–2 weeks [36]. Since stem cells are the only cells in flatworms that are capable of proliferation [37], the primary cell system reflects the proliferative capacity of the parasite's stem cells. Using this system, we have already investigated the influence of excretory/secretory (E/S) products of the early developing parasite larvae on host immune cells [28]. In this previous study, a central role of larval E/S products in host immunomodulation by *E. multilocularis* was demonstrated. However, nothing is known so far on the role of parasite E/S products in the onset of the Th1 immune response that governs the early phase of the infection by *E. multilocularis*.

In the current study, a new *E. multilocularis* E/S product, EmTIP, with homology to human and murine T-cell Immunomodulatory Proteins [38] was identified. EmTIP was expressed by primary cells and metacestode vesicles, but only found to be present in primary cell E/S products.TIP homologues are widely expressed by eukaryotes, suggesting an important function in fundamental biological processes [39]. However, except for a single study on T-cell immunomodulatory activities [38], their function is still unknown. As yet, human and murine TIPs have primarily been shown to elicit a strong IFN-γ release by murine and human T-cells, accompanied by elevated levels of IL-10 and TNF-α, translating into a protective effect in a murine model of Graft-versus-host disease [38]. Herein, we demonstrate that the TIP homologue of *E. multilocularis* is important for the proliferation and transition of parasite primary cells into metacestode vesicles and promotes IFN-γ release by murine CD4+T-cells *in vitro*. The characterization of EmTIP, a candidate target for anti-AE therapy, is reported.

## Materials and Methods

### Animals and ethics statement

C57BL/6 mice were purchased from Charles River and housed at the local animal facility of the Institute for Hygiene and Microbiology at least 1–2 weeks before experimentation as were Mongolian jirds (*Meriones unguiculatus*). Animal handling, care and subsequent experimentation were performed in compliance with German (*Deutsches Tierschutzgesetz*, *TierSchG*, version from Dec-9-2010) and European (European directive 2010/63/EU) regulations on the protection of animals. Ethical approval of the study was obtained from the local ethics committee of the government of Lower Franconia (*Regierung von Unterfranken* 55.2-2531.01-31/10).

### Parasite material

*E. multilocularis* primary cells and metacestode vesicles were isolated, separated from host contaminants and maintained in axenic cultures as previously described [40]. For the collection of E/S products, axenically maintained parasite larvae were washed thrice in 1× PBS and resuspended in collection medium DMEM_10_redox; i.e. dulbecco's modified eagle's medium, 4.5 g glucose/L (Gibco) supplemented with 10% fetal bovine serum Superior (Biochrom AG), 100 µg/ml penicillin;streptomycin (Biochrom AG), 20 µg/ml levofloxacin (Sanofi-Aventis), 143 µM ß-mercapthoethanol (Sigma-Aldrich), 10 µM bathocuproine disulfonic acid (Sigma) and 100 µM L-cysteine (Sigma) under axenic conditions (i.e. sealed in Nitrogen filled Ziploc freezer bag and placed in a 5% CO_2_ incubator at 37°C). After 48 hours of culture, the supernatants containing the larval E/S products were collected and filtered through a 0.2 µm sieve (Filtropur S filter, SARSTEDT). The total amount of proteins per supernatant was determined using the bicinchoninic acid assay (ThermoScientific) and normalized by addition of ddH2O prior to storage at −80°C until use.

### Identification, cloning and analysis of the Em*tip* gene and cDNA

For identification, a previously established *E. multilocularis* cDNA library for trans-spliced transcripts [41] was randomly sequenced and found by tblastn analyses to contain a recombinant clone encoding a homolog of the human T-cell Immunomodulatory Protein [38].

For RNA isolation and cDNA synthesis, total RNA was isolated from *in vitro* cultivated parasite primary cells and metacestode vesicles by guanidinium thiocyanate-phenol-chloroform extraction using Tri reagent (Invitrogen) according to the manufacturer's instructions. Due to the fluid-containing structure of metacestode cysts, the protocol was slightly modified. Briefly, a maximum of 5 cysts were ruptured using a sterile needle (23G, Dispomed Witt oHG). The released cyst fluid was removed upon centrifugation at 400 g for 3 minutes at RT, then the pellet was resuspended in 1 ml Tri reagent (Invitrogen) and subsequently processed as per the manufacturer's guidelines. The isolated RNA (1 µg) was reverse transcribed into cDNA using a reverse transcription Kit (Qiagen) in a total volume of 20 µl according to the manufacturer's instructions

Amplification and cloning: The 5′and 3′ends of the coding sequence were used to design primers for full length amplification, namely Emtip_Dw (5′-CCT TGC AGT TTT GTA TGA AAA TG-3′) and Emtip_Up (5′-GAT CAT TCG ACC TTC TAC ATT GC-3′). cDNA from the parasite larvae was used as a template in a polymerase chain reaction with the designed primer pair Emtip_Dw/Emtip_Up and a high fidelity polymerase chain reaction (New England Biolabs). Resulting amplicons were sub-cloned into the pDrive cloning vector (Qiagen) and sequenced in both directions.

*In silico* analyses: Sequence similarities between the deduced amino acid sequence of EmTIP and other members of the TIP family were determined through multiple sequence alignments using the BioEdit BLOSUM62 similarity matrix (http://www.mbio.ncsu.edu/bioedit/bioedit.html). The domains of T-cell immunomodulatory proteins were predicted using SMART online tool (http://smart.embl-heidelberg.de/) with default settings.

### RT-PCR

Prior to the analysis of Em*tip* expression by *E. multilocularis* larvae, the Em*tip* cDNA was aligned to its genomic locus and the exon-intron boundaries defined. For unambiguous RT-PCR, the intron-flanking and Em*tip*-specific primer pair 5′-GAA ACG TTG AAA CAG ATC G-3′ (FG-GAP_Dw) and 5′-GCT TAT GTT TCC GAG CTT G-3′ (FG-GAP_Up) was designed. 1 µl of *E. multilocularis* primary cell and metacestode vesicle cDNA preparations, reverse-transcribed (Qiagen) from 1 µg of RNA, was amplified using the Em*tip*-specific primer set FG-GAP_Dw/FG-GAP_Up and the following amplification conditions: 30 sec denaturation at 94°C, 30 sec annealing at 55°C, and 30 sec extension at 72°C for 30 cycles. PCR products were separated by agarose gel electrophoresis (1%) and visualized under UV light after staining with ethidium bromide.

### Immune serum production and purification of anti-EmTIP antibody

Production of an anti-EmTIP serum: For the production of polyclonal antibodies, EmTIP was expressed in the bacterial pBAD/topo thio fusion expression kit (Invitrogen). An *in silico* predicted, immunodominant portion (http://tools.immuneepitope.org/ tools/bcell/iedb_input) of Em*tip* (from position 111 to 1119 bp of the coding sequence) was sub-cloned in pBAD/topo thio fusion following amplification with the primer pair AbTIP_dw (5′-GCT GAT TTG GCC GCT TTT G-3′) and AbTIP_Up (5′-CTG AGA TTC AAC TCC AGG CAA AAG-3′). The Thioredoxin- fusion protein (Thio-EmTIP) with histidine tag, expressed in *E.coli* Top10 by Arabinose-induction (2 g/L; 4 hours), was purified on nickel-nitrilotriacetic acid resin (Invitrogen) according to the manufacturer's protocol. The purified Thio-EmTIP was then processed on centrifugal filter units (Millipore) against sterile PBS (1X) before quantification using the BCA assay (ThermoScientific). Inbred rabbits (1 year old) were intradermally immunized repetitively with the Thio-EmTIP antigen suspensions (ImmunoGlobe Antikörpertechnik GmbH; project IG1149) generating 50 ml of polyclonal antiserum. An aliquot of pre-immune serum was used as specificity reference for the polyclonal anti-EmTIP immune serum.

Affinity purification of the anti-EmTIP serum: 200–500 µg of purified Thio-EmTIP antigen was run on SDS PAGE and then blotted onto a nitrocellulose membrane. The band was visualized by ponceau S staining (Sigma-Aldrich) and excised prior to blocking in 5% skimmed milk in TBST (i.e. 5 g of skimmed milk in 100 ml of 1 xTris Buffer Saline supplemented with 0.1% Tween 20) for 1 hour at room temperature on a rocking plate. The nitrocellulose strip was then incubated overnight at 4°C with 500 µl of anti-Thio-EmTIP antiserum on a rotating wheel. Next, the strip was sequentially washed in 0.15 M NaCl, 1× PBS, then the bound antibodies were eluted with 0.2 M glycine solution supplemented with 1 mM EGTA at pH 2.5. The eluted anti-EmTIP antibody solution was neutralized to pH 7 with Tris solution (1 M), then diafiltered on centrifugal filter units (Millipore) against sterile PBS (1X) and filtered (0.2 µm; Millipore) before quantification of the protein content using the BCA assay (ThermoScientific) according to the manufacturer's instructions and, finally, stored at −20°C until use. In parallel, a whole γ class Immunoglobulin (IgG) solution from naïve rabbit (Santa Cruz Biotechnology, inc.) was similarly resuspended in 0.2 M glycine solution supplemented with 1 mM EGTA at pH 2.5, neutralized, diafiltered, filtered (0.2 µm; Millipore) and quantified for protein content (ThermoScientific) before storage at −20°C.

### Recombinant expression of EmTIP in HEK-293T cells

Human embryonic kidney- (HEK-) 293T cells were transiently transfected with an eukaryotic EmTIP-expressing vector system. Briefly, the full-length coding sequence of Em*tip* with or without a c-terminal stop codon (adding a short myc-tag to the C-terminus) was sub-cloned into the expression vector pSegTag2 Hygro A (Invitrogen) according to the manufacturer's instructions, yielding respectively the pSecTag2-Em*tip* and pSecTag2-Em*tip*_c-Myc vector constructs. HEK-293T cells were transfected with the Em*tip* constructs, pSecTag2 Hygro A vector as a negative control or with ddH2O as a mock control. Transfections were performed using linear polyethyleneimine (25 kDa, Sigma) as instructed by the manufacturer. All transfections were performed in petri dishes (92×16 mm [Ø×height], SARSTEDT) seeded with cells 16 hours prior to transfection (3×10^6^ cells/dish). 24 hours post-transfection, the culture supernatant was replaced by fresh medium. After another 24 hours, the supernatant was collected and the presence of recombinant EmTIP (rEmTIP) was verified by immunoprecipitation followed by Western blot. The harvested supernatants of transfected HEK-293T cells were filtered over a bottle top filter (SARSTEDT), normalized for the total protein content (BCA protein assay kit, ThermoScientific) and stored as aliquots at −80°C until use.

### Immunodetection

Protein lysates were obtained from primary cell cultures, metacestode vesicles, and HEK-293T cells transfected with pSecTag2, pSecTag2-Em*tip* and pSecTag2-Em*tip*_c-Myc via treatment with 2× STOPP mix (2 ml 0.5 M Tris-HCl pH 6.8, 1.6 ml glycerol, 1.6 ml 20% SDS, 1.4 ml H2O, 0.4 ml 0.05% (w/v) bromophenol blue, 7 µl ß-mercaptoethanol per 100 µl) for 10 min at 100°C.

E/S products of *E. multilocularis* primary cell cultures and metacestode vesicles as well as supernatant of HEK-293T cells transfected with pSecTag2, pSecTag2-Em*tip* and pSecTag2- Em*tip*_c-Myc were first probed by immunoprecipitation with anti-EmTIP antibody (for the parasite E/S products, pSecTag2 and pSecTag2-Em*tip*- transfected HEK-293T supernatants) or anti-c-Myc antibody (Santa Cruz, for pSecTag2 and pSecTag2- Em*tip*_c-Myc -transfected HEK-293T supernatants). More precisely, anti-EmTIP antibody (1∶20) or anti-c-Myc (2 µg) was captured on protein G agarose (Cell Signaling Solutions) according to the manufacturer's instructions. The antibody-coated protein G agarose beads were packed into a column and used to capture rEmTIP proteins (i.e. rEmTIP and rEmTIP_myc) from supernatants. The captured antigens were eluted in 2× STOPP, mixed and boiled for 10 min at 100°C.

Immunodetection: To assess EmTIP expression/secretion by *E. multilocularis* larvae and transfected HEK-293T cells, the above-obtained somatic protein extracts and immunoprecipitated antigen preparations were separated by SDS-PAGE, electroblotted (Western blot), and probed with anti-EmTIP antiserum (1∶10,000) as well as pre-immune serum (1∶10,000). Control (pSecTag2-) and pSecTag2-Emtip_c-Myc-transfected HEK-293T supernatants were probed with anti-c-Myc antibody (1∶1000). Affinity purified HRP-conjugated anti-rabbit IgG (Jackson ImmunoResearch) and Affinity purified HRP-conjugated anti-mouse IgG (Jackson ImmunoResearch) were used to detect bound rabbit and mouse antibodies respectively. The secondary antibodies were detected using ECL reagents (Thermoscientific) as per manufacturer's instructions.

### Immunolocalization

*E. multilocularis* primary cell cultures and metacestode vesicles axenically maintained *in vitro* [40] as well as non-infected and *E. multilocularis*-infested liver tissue from jirds were fixed and cut by a microtome into sections of 4 µm. Briefly, the samples were fixed in 2% paraformaldehyde (in 1× PBS) and washed at 4°C overnight in 1× PBS containing 6.8% sucrose. The tissues were then dehydrated in 100% acetone for 1 hour at 4°C prior to embedding using a glycol methacrylate embedding kit (Heraeus Kulzer Technik) according to the manufacturer's instructions. Next, using a rotary microtome, 4 µm sections were prepared and stained sequentially with purified anti-EmTIP antibody then peroxidase-coupled anti-rabbit-IgG (Jackson ImmunoResearch) followed by haematoxilin counterstaining according to the manufacturer's instructions (Heraeus Kulzer Technik). The sections were examined by light microscopy and images captured with a digital camera.

### *In vitro* neutralization assay

Rabbit polyclonal anti-EmTIP antibody, obtained as described above by enrichment of anti-EmTIP immune serum over nitrocellulose-bound recombinant thio-EmTIP, was used for *in vitro* binding of EmTIP in *E. multilocularis* primary cell cultures. Primary cells were isolated as previously described [28] and adjusted to a turbidity of OD600 nm 14/ml in culture medium DMEM10redox. All experiments were performed in a flat-bottom 96 well plates (Nunc, Thermo Scientific) with 10 µl of primary cells suspension per well in a final volume of 100 µl supplemented or not with different concentrations of purified anti-EmTIP antibody. As control, total IgG from naïve rabbit (Santa Cruz) resuspended in the anti-EmTIP antibody carrier solution was used. *In vitro* culture of primary cells without treatment was used as positive control. For the cultivation of primary cells, the plates were sealed in ziploc freezer bags filled with nitrogen before incubation in a CO_2_ incubator at 37°C for 36 hours.

Next, 10 µM BrdU solution (BrdU Cell proliferation Elisa kit; Roche Applied Science) was added to the cultures and the cells were further incubated for 4 additional hours. The cells were subsequently fixed and the amount of incorporated BrdU was revealed after DNA denaturation, probing with a peroxidase conjugated anti-BrdU monoclonal antibody and finally colorimetrical detection with tetra methyl-benzidine (peroxydase substrate) according to the manufacturer's instructions (BrdU Cell proliferation Elisa kit, Roche Applied Science). In parallel, to identify any effects of anti-EmTIP antibody on general cell proliferation, 0.1×10^4^ Rat hepatoma cells were seeded in 100 µl of culture medium with or without anti-EmTIP antibody and the proliferation rate was assessed by BrdU incorporation as outlined above.

For the in vitro transition of primary cells into metacestode vesicles, 100 µl of primary cell suspension in DMEM10redox was seeded in a 48 well plate format supplemented or not with anti-EmTIP antibody in flat-bottom 48 well plates (Thermo Scientific). The plates were sealed in ziploc freezer bags filled with nitrogen before incubation in a CO_2_ incubator at 37°C. The medium was replaced with fresh DMEM10redox (control), total rabbit IgG- or anti-EmTIP- supplemented DMEM10redox every 48 hours. At day 8, the DMEM10redox medium was completely aspirated and fully replaced with medium that had been pre-conditioned by rat hepatoma cells (A4 medium) to support the *de novo* formation of metacestode vesicles from primary cell cultures [40]. The cultures were then further kept with frequent A4 medium change (twice per week). At day 22, when cyst generation was apparent, clusters of primary cells were dissociated by gentle pipetting of the supernatant and flushing of cell clusters with a 1-mL pipette to release hidden cysts. The total number of cysts per well was then determined by microscope-aided counting.

### T-cell stimulation assays

CD4+ T-cells were isolated from spleen and lymph nodes of healthy C57BL/6 mice (6–8 weeks old) using a T-cell negative selection kit (mouse CD4+ T-cell enrichment kit, Stem Cell Technologies) to >90% purity according to the manufacturer's instructions. The CD25− fraction was further enriched using column-based cell isolation (Miltenyi Biotec) equipped with a suitable magnet-based cell separator achieving >90% purity for CD4+CD25− T-cells. Next, 2×10^5^ CD4+CD25− T-cells were seeded in a 24-well tissue culture plate (Flat bottom, SARSTEDT) that had been coated with anti-CD3 (0.1 µg/ml, eBioscience) overnight at 4°C. The cell suspension was supplemented with 5 µg/ml anti-CD28 antibody (eBioscience). rEmTIP-containing and control HEK-293T cell supernatants were then added to T-cell cultures. Secretions of the parasite larvae were either directly added to T-cell cultures or first supplemented and pre-incubated for 30 minutes at room temperature with 30 µg/ml of rabbit IgG or purified anti-EmTIP antibody before T-cell stimulation. 72 hours later, the culture supernatants were collected for determination of IL-10 and IFN-γ release by T-cells using ELISA kits (BD Biosciences) with detection limits of 19 pg/ml and 0.762 pg/ml respectively according to the manufacturers' instructions. Alternatively, Brefeldin A (5 µg/ml; Sigma) was added during the final 6 h of stimulation and the cells were harvested for intracellular FACs analysis. Briefly, the cells were stained for surface markers (CD4-PE, BD Biosciences) in ice-cold PBS supplemented with 0.1% BSA and 0.1% sodium azide, followed by fixation in 2% formaldehyde and permeabilization in perm buffer (0.5% saponin in PBS) and then stained in perm buffer for intracellular IFN-γ (IFN-γ-FITC, BD Biosciences). Samples were measured at a flow cytometer (BD) and data were analyzed with a flow cytometry analysis software (TreeStar).

### Statistical analyses

All results were expressed as mean ± standard deviation (SD). Differences observed between groups were evaluated using the Wilcoxon/Mann-Whitney U test, a nonparametric test that does not assume normality of the measurements (it compares medians instead of means). Values of p<0.05 were considered statistically significant. Statistical analyses were performed with a statistical software analyzing package (GraphPad Software).

### Accession numbers

Sequence data of *E. multilocularis* T-cell Immunomodulatory Protein reported in this manuscript are available from GenBank (http://www.ncbi.nlm.nih.gov/Genbank) under accession number HF912277. Other GenBank accession numbers of genes and sequences used in this study include: Human TIP (Q8TB96); mouse TIP (Q99KW9) and *E. granulosus tip* (EgrG_000440000) on GeneDB (http://www.genedb.org)

## Results

### Characterization of the Em*tip* cDNA and gene

**Figure 1:**
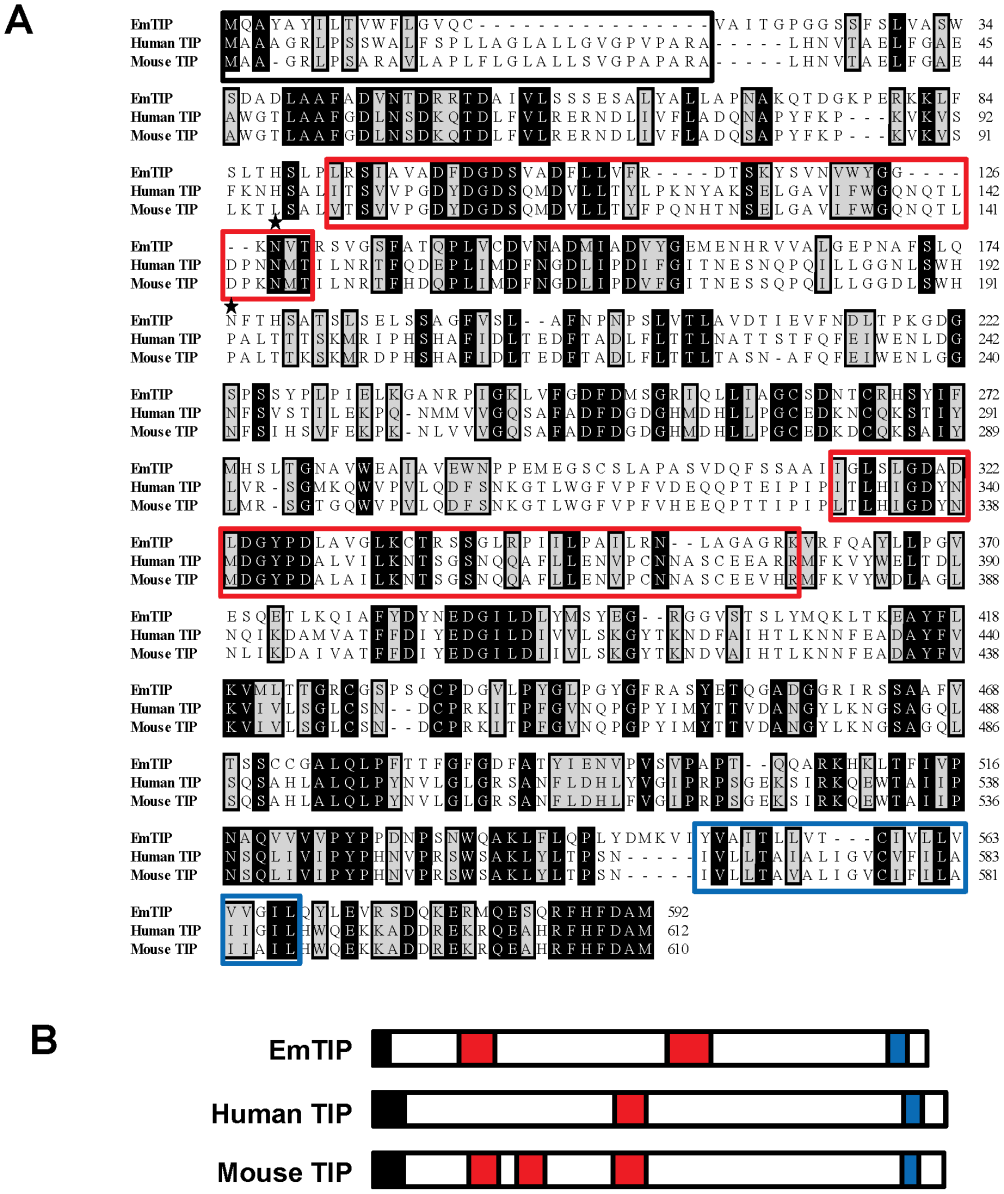
EmTIP: a T-cell immunomodulatory protein homologue from *E. multilocularis*. (**A**) Alignment of EmTIP with human and mouse TIPs. Bioedit software, version 7.0.9, was used to align the amino acids sequences. Accession numbers for the sequences are as follows: EmTIP, HF912277; human TIP, Q8TB96; mouse TIP, Q99KW9. Identical residues are displayed in white on black background, biochemically similar residues in black on grey background. Gaps introduced to maximize the alignment are represented by dashes. Numbers at the end of each line correspond to the amino acid numbers in each respective sequence. EmTIP has 36% identity/48% similarity with Human TIP and 34% identity/42% similarity with Mouse TIP. The N-terminal signal sequences are shown in an open solid black box. Two FG-GAP repeats within EmTIP as predicted by SMART (http://smart.embl-heidelberg.de/) are shown within open solid red boxes. The C-terminal transmembrane domains are depicted in an open solid blue box. (**B**) Comparative structural architecture of *E. multilocularis*, human and mouse T-cell immunomodulatory proteins. Red boxes: signal sequences; Grey boxes: FG-GAP repeats; Blue boxes: Transmembrane domains. Sequence accession numbers are as described in (**A**), with structural architecture predicted with SMART (http://smart.embl-heidelberg.de/).

In previous investigations on the mechanism of trans-splicing in *E. multilocularis* [41], we isolated a cDNA clone that apparently encoded a protein with significant homology to human TIP. Since human TIP has been shown to influence cytokine production in T-cells [38], and considering immunomodulatory activities of *Echinococcus* larvae [4], [9], [28], [30], [42], we further investigated the parasite factor and named it EmTIP (*Echinococcus multilocularis* T-cell Immunomodulatory Protein homologue) (Genbank accession number HF912277). The corresponding cDNA sequence harbors an *Echinococcus multilocularis* spliced leader sequence at its 5′end (Fig. S1), suggesting that the Em*tip* mRNA is processed via trans-splicing. The full-length Em*tip* cDNA comprises 1,974 bp (excluding the polyA-tail) and encodes a protein of 592 amino acids (Fig. S1) with a hydrophobic region at the N terminus, indicating the presence of a signal peptide. The predicted gene product, EmTIP, shows moderate but significant sequence homology to human and mouse TIP-orthologs (36% and 34% identity, respectively; Fig. 1A). It contains two N-glycosylation sites, two FG-GAP (phenylalanyl-glycyl and glycyl-alanyl-prolyl) repeats and a transmembrane domain (Fig. S1). Thus, overall, EmTIP shows a structural architecture common to human and mouse TIPs (Fig. 1B).

The genomes of *E. multilocularis* and its close relative *E. granulosus* (dog-tapeworm) have recently been sequenced [43]. When using the Em*tip* cDNA sequence as a query, we identified the respective genomic locus as a single copy gene on scaffold 7614 (gene designation: EmuJ_000440000). The Em*tip* gene comprises 10 exons, separated by 9 introns. Extensive BLAST searches revealed that no other genes with significant homology to Em*tip* were present on the *E. multilocularis* genome. On the *E. granulosus* genome we also identified an orthologous gene, Eg*tip* (EgrG_000440000) that encodes a protein with 98.5% identity to EmTIP. Hence, orthologs to human TIP are apparently encoded by the genomes of both important human parasitic *Echinococcus* species.

### EmTIP expression pattern

**Figure 2:**
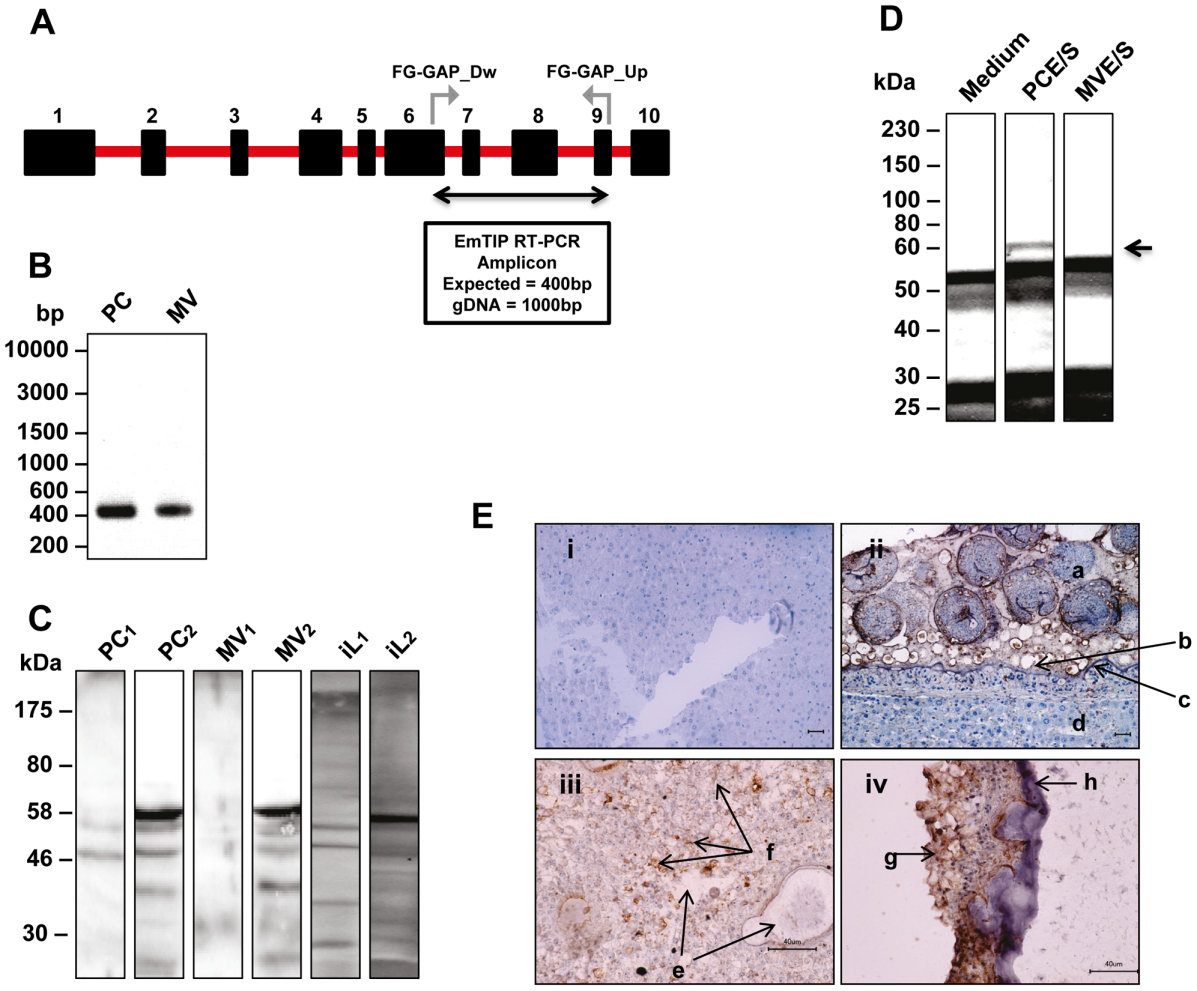
Expression pattern of EmTIP and localization within *E. multilocularis* larvae. (**A**) RT-PCR strategy for the amplification of the *emtip* transcript. Shown is an intron (red line)-exon (black boxes) arrangement of the EmTIP genomic locus. A 400 bp product specific for *emtip* was amplified using the primers FG-GAP_Dw and FG-GAP_Up spanning from exons 6–9. (**B**) *E. multilocularis* larvae (PC and MV) were separately used for qualitative assessment of *emtip* expression. 1 µl of cDNA of each larva was used as template for PCR with a high fidelity DNA polymerase (High-Fidelity DNA Polymerase, New England Biolabs). 2 µl of each PCR amplicon were resolved on a 1.5% agarose gel and stained with Ethidium bromide prior to visualization under a UV transilluminator. (**C**) Western blots of *E. multilocularis* larvae lysates probed with pre-immune rabbit serum (1) or anti-EmTIP immune-serum (2) followed by ECL detection and autoradiography. Whole larvae lysates (PC and MV) and *E. multilocularis*-infected jird liver (iL) were probed with pre-immune rabbit serum (1) or rabbit anti-EmTIP immune-serum followed by ECL detection and autoradiography. (**D**) Western blot of E/S products from *E. multilocularis* larvae after immunoprecipitation. Samples were probed with affinity-purified anti-EmTIP antibody followed by ECL detection and autoradiography. The positions of the molecular mass markers (in kilodaltons) are shown on the left. Arrow indicates the position of EmTIP around 58 kDa in primary cell E/S products. The blot is representative of two experiments with different biological isolates with similar results. (**E**) Staining with affinity-purified anti-EmTIP antibody followed by peroxydase-coupled anti-rabbit-IgG (Jackson ImmunoResearch) against haematoxilin counterstain of representative tissue sections from healthy jird liver (**i**), infected jird liver (**ii**), *in vitro* cultivated *E. multilocularis* primary cells (**iii**) and metacestode vesicles (**iv**). Note the EmTIP signal within the parasite intercellular spaces. Legend: a, *E. multilocularis* intra-cystic compartment with protoscoleces; b, germinal layer; c, laminated layer; d, periparasitic jird liver tissue; e, central cavities surrounded by aggregates of *E. multilocularis* primary cells as previously defined [36]; f, EmTIP signal; g, germinal layer; h, laminated layer. Scale bars: 40 µm.

To determine the expression patterns of Em*tip*, reverse transcriptase–polymerase chain reaction (RT-PCR) was performed on cDNA from axenically maintained primary cell cultures and metacestode vesicles, the two larval stages that are relevant for the infection of the intermediate host [28]. Em*tip* transcripts could be detected in both larval stages (Fig. 2A, B). Western blot analyses using a polyclonal antibody against recombinant thio-EmTIP were then used to determine the protein expression profile. The anti-EmTIP serum recognized a single 58-kDa protein in primary cell preparations, metacestode vesicles and liver extracts from infected jirds (Fig. 2C), which correlates well with the predicted molecular mass of EmTIP (64 kDa). Interestingly, when examined for EmTIP secretion by immunoprecipitation with an affinity-purified anti-EmTIP antibody (Fig. S2), a band of the expected size was detected in E/S products of *E. multilocularis* primary cells, but not in those of metacestode vesicles (Fig. 2D).

To investigate the localization of EmTIP within the parasite, we performed immunohistochemical detection on sections of each larva. The affinity-purified anti-EmTIP antibody (Fig. S2) detected EmTIP mainly in the inter-cellular space and the surface of cells in primary cell culture aggregates and metacestodes (Fig. 2E). Poor association of EmTIP was seen with intracellular structures in both larvae or in parasite tissue present in infected jirds (Fig. 2E). No signal was obtained for liver tissue of non-infected jirds (Fig. 2E).

### EmTIP is necessary for *E. multilocularis* stem cell proliferation and the formation of metacestode vesicles

**Figure 3:**
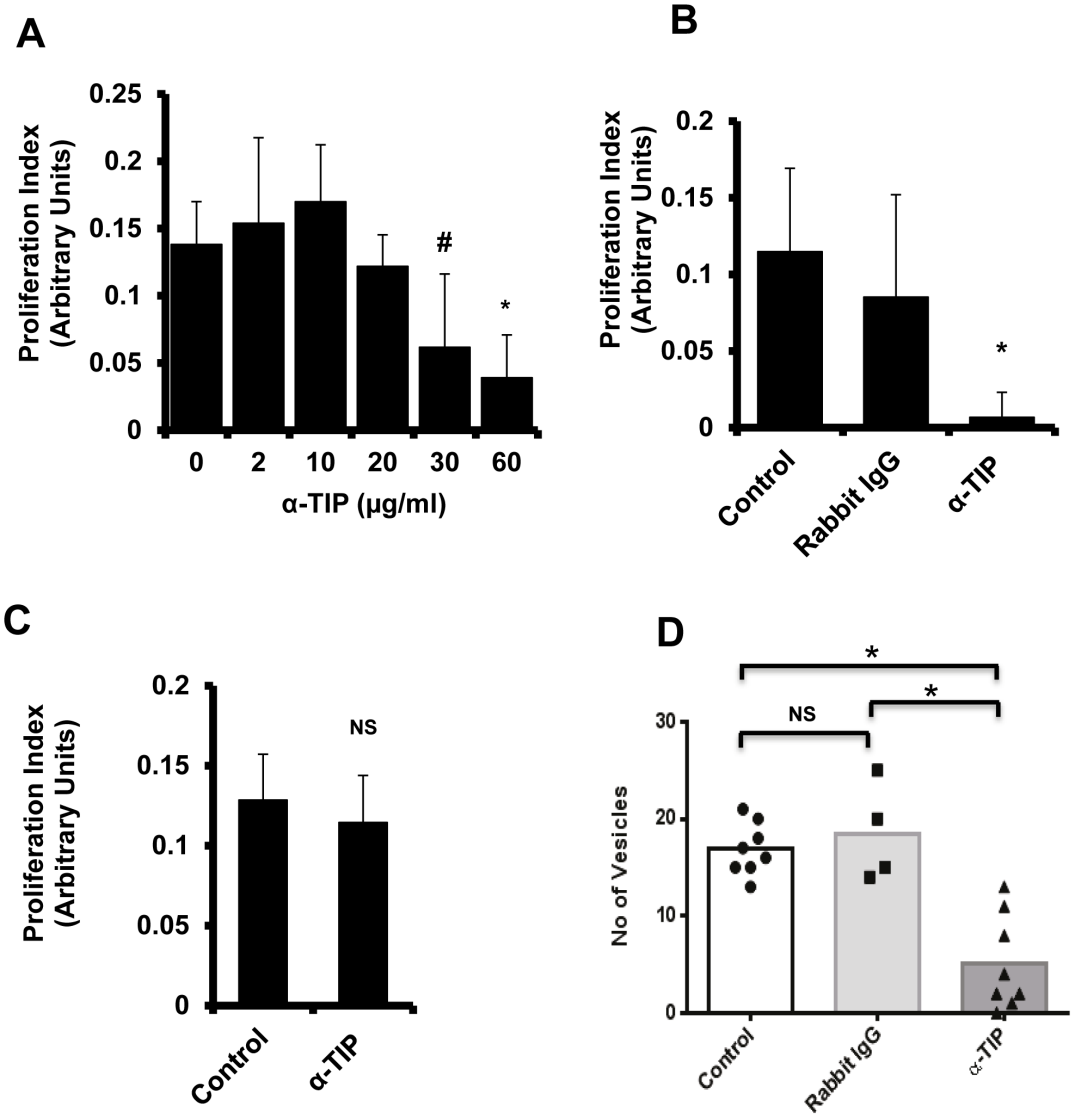
Anti-EmTIP antibody impairs *E. multilocularis* stem cell proliferation and metacestode vesicle formation *in vitro*. (**A**) Dose-response curve of the antiproliferative effect of EmTIP inhibition using affinity-purified anti-EmTIP antibody on *E.multilocularis* primary cell cultures. *E. multilocularis* stem cell proliferation was assessed by measuring the level of BrdU incorporation (see material and methods for details of the BrdU cell proliferation assay) here expressed as proliferation index. (**B**) 60 µg/ml of anti-EmTIP antibody limits *E. multilocularis* stem cell proliferation whereas an equal amount of total rabbit IgG failed to do so. (**C**) Effect of anti-EmTIP antibody (60 µg/ml) on rat hepatoma cell line proliferation. (**D**) Effect of anti-EmTIP antibody vs. rabbit IgG on the *de novo* formation of *E. multilocularis* metacestode vesicles from primary cell cultures. (**A, B, C**) Bars represent mean ± SD for 4–6 replicates of two independent experiments. (**D**) Bars stand for mean levels. Data represent 4–8 biological primary cell isolates assayed individually. ^#^, statistical trend *p<0.1*;*, statistical significance *p<0.05*; NS, not significant *p>0.1*.

To assess the role of EmTIP in *E. multilocularis* larval development, an affinity-purified anti-EmTIP antibody (Fig. S2) was used. When cultivated in the presence of this antibody, *E. multilocularis* stem cell proliferation was significantly impaired in a dose-dependent manner as assessed by BrdU incorporation assays (Fig. 3A). To exclude the possibility of a non specific effect of the anti-EmTIP antibody, an amount of total rabbit IgG equivalent to the most inhibitory dose of anti-EmTIP antibody (60 µg/ml, Fig. 3A) was used, but revealed no inhibition of *Echinococcus* stem cell proliferation (Fig. 3B). In addition, a similar amount of anti-EmTIP antibody did not inhibit the proliferation of rat hepatoma cells (Fig. 3C), indicating that the antibody preparation did not exhibit general cytotoxic effects.

Using the primary cell culture system, we also investigated the effect of the anti-EmTIP antibody on the *de novo* formation of metacestode vesicles. As shown in Fig. 3D, we observed a significantly reduced rate of metacestode vesicle formation in anti-EmTIP supplemented primary cell cultures after 22 days of incubation (p<0.05). Taken together, these data suggest that treatment of *E. multilocularis* primary cell cultures with anti-EmTIP antibody significantly affects parasite stem cell proliferation and larval development.

### EmTIP promotes IFN-γ release by host CD4+ T-cells

**Figure 4:**
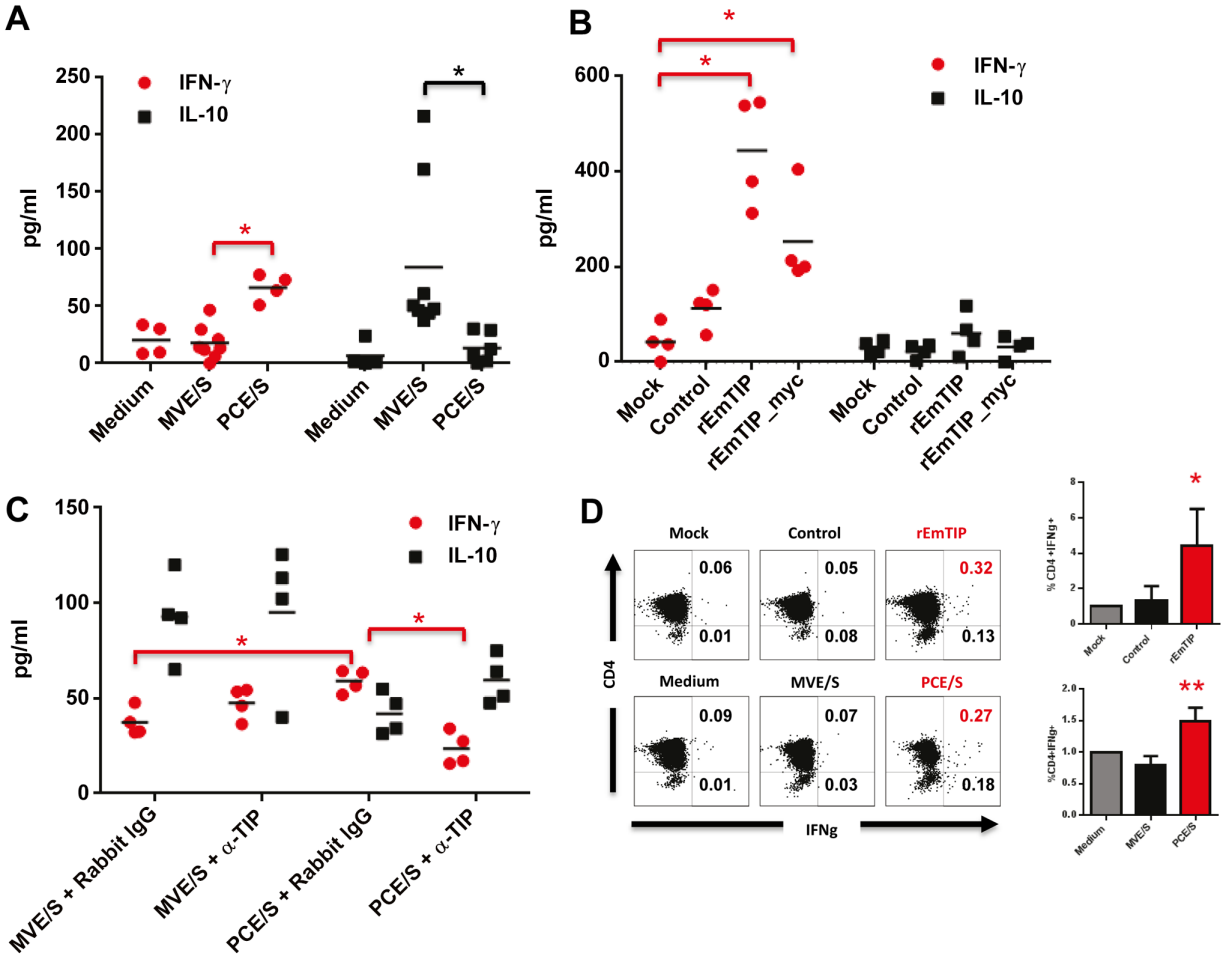
Elevated secretion of IFN-γ by CD4+ T-cells activated in the presence of EmTIP *in vitro*. (**A**) Freshly isolated CD4+CD25− T-cells (2×10^5^/ml) were stimulated with α-CD3 (0.1 µg/ml) and α-CD28 (5 µg/ml) in the presence of E/S products of *E. multilocularis* primary cells (PCE/S) or metacestode vesicles (MVE/S). Three days later, the T-cell supernatants were collected and probed for IFN-γ and IL-10 concentrations by Elisa. (**B**) Freshly isolated CD4+CD25− T-cells (2×10^5^/ml) were stimulated with α-CD3 (0.1 µg/ml) and α-CD28 (5 µg/ml) in medium supplemented with normalized supernatants of Mock-, control- (pSecTag2), Em*tip*- (pSecTag2-Em*tip*) or Em*tip*_myc (pSecTag2-Em*tip*_myc)- transfected HEK-293T cells. Three days later, the T-cell supernatants were collected and probed for IFN-γ and IL-10 concentrations by Elisa. (**A, B**) Individual results are displayed. Bars represent the mean from 4–8 independent biological replicates assessed within 3–4 independent experiments. (**C**) Freshly isolated CD4+CD25− T-cells (2×10^5^/ml) were stimulated with α-CD3 (0.1 µg/ml) and α-CD28 (5 µg/ml) in the presence of conditioned medium of primary cells (PCE/S) or metacestode vesicles (MVE/S) supplemented with total rabbit IgG or purified anti-EmTIP (anti-TIP) antibody at 30 µg/ml. Three days later, the T-cell supernatants were collected and probed for IFN-γ and IL-10 concentrations by Elisa. Individual results are displayed. Bars represent the mean from experiments with 4 biological replicates. (**D**) Freshly isolated CD4+CD25− T-cells (2×10^5^/ml) were stimulated with α-CD3 (0.1 µg/ml) and α-CD28 (5 µg/ml) in medium supplemented with normalized supernatants of parasite larvae (PCE/S or MVE/S) or transfected HEK-293T cells (Mock, Control or rEmTIP). At day 3 of culture, 5 µg/ml Brefeldin A was added for an additional 6 hours. Then the cells were harvested and the intracellular IFN-γ levels were determined by flow cytometry. Numbers in upper gates indicate the percentage of IFN-γ+ CD4+ cells whereas the lower gate shows the percentage of IFN-γ+ CD4− cells. One representative experiment of 3 conducted with similar results is shown (Left) and data from all three experiments are summarized as bar graphs (Right). * (p<0.05), ** (p<0.01) (**D**) Bars represent mean ± standard deviations from three independent experiments with 3–7 biological replicates.

Next, to assess the differential effects of primary cell vs. metacestode E/S products on T-cells, naive murine CD4+ T-cells were activated in the presence of secretions of each larva. When analyzing for the resulting cytokine profile, we observed a pronounced ability of primary cell E/S products to elicit IFN-γ but not IL-10 release when compared to those of metacestode vesicles (Fig. 4A).

Given that EmTIP could only be detected within the primary cell E/S products and not those of metacestode vesicles (Fig. 2D), we investigated the role of EmTIP in the IFN-γ release by CD4+ T-cells when exposed to primary cell E/S products. To this end, EmTIP was recombinantly expressed in HEK-293T cells, the transfected cells were probed for EmTIP expression, and their supernatant was analyzed for rEmTIP (rEmTIP) release (Fig. S3). rEmTIP proteins, as present in the supernatant of transfected HEK-293T cells (Fig. S3C, E), were added to CD4+ T-cell cultures during activation and the resulting cytokine profile was determined. We noted an elevated release of IFN-γ, but not IL-10, in rEmTIP-treated T-cell cultures when compared to control-treated T-cell cultures (Fig. 4B). We noted a similar ability of rEmTIP_myc to promote IFN-γ release by murine CD4+ T-cells (Fig. 4B).

To ascertain the role of EmTIP in the promotion of IFN-γ release by CD4+ T-cells, we pre-incubated the parasite E/S products with anti-EmTIP antibody before addition to T-cell cultures. We observed a significant reduction of IFN-γ release by T-cells when primary cell secretions were supplemented with anti-EmTIP (Fig. 4C) confirming that EmTIP is important for the ability of *E. multilocularis* primary cell secretions to promote IFN-γ release by T-cells.

Finally, to confirm that CD4+ T-cells were the true source of IFN-γ release in our cultures, intracellular flow cytometric analysis was performed on treated T-cell cultures. It appeared that CD4+ T-cells were the principal source of the IFN-γ release promoted by primary cell E/S products or rEmTIP (Fig. 4D).

Our data, thus collectively, suggest that EmTIP, secreted by primary cells of *E. multilocularis*, promoted IFN-γ release by murine CD4+ T-cells *in vitro*.

## Discussion

IFN-γ and its associated effects dominate the very early post-oncospheral phases of an infection with *E. multilocularis* [3], [29], [30] and are potentially deleterious to parasite larvae [12], [13], [15], [25], [44]. The resulting Th1 immune response is progressively suppressed as the metacestodes are formed and disseminate within the host [45]. The mechanisms mediating the early onset of host protective Th1 responses against *E. multilocularis* larvae are still not known. In the present study, we report on a parasite factor with significant homology to the human T-cell immunomodulatory protein (TIP). Since human TIP is known to promote the release of IFN-γ, TNF-α and IL-10 by T-cells [38], we characterized and functionally assessed the parasite TIP homologue, EmTIP, regarding its ability to promote the release of these cytokines by CD4+ T-cells. The corresponding gene, Em*tip*, codes for an atypical FG-GAP repeat containing protein, EmTIP. We showed that EmTIP is expressed by the larval stages that infect the intermediate host and that it is released in soluble form by *Echinococcus* primary cells, representing the early invading stage for the intermediate host. We also showed that EmTIP localized to the inter-cellular space of the parasite larvae and that an antibody directed against EmTIP limited the proliferation of the parasite stem cells and restrained their ability to form metacestode vesicles *in vitro*. Finally, we found that EmTIP instructed murine CD4+ T-cells to release IFN-γ but not IL-10 *in vitro*. Taken together, these findings suggest that EmTIP is a parasite factor important for the early phase of *E. multilocularis* larval development and at the same time capable of promoting IFN-γ release by CD4+ T-cells.

Although an earlier study associated mammalian TIP with a protective effect in a graft-versus-host model, resulting in the release of a complex cytokine profile (IFN-γ, IL-10 and TNF-α) by T-cells [38], the precise cellular and/or physiological function of the TIP protein family is elusive so far. TIP orthologs are widely conserved throughout the animal kingdom and are present in numerous invertebrates, suggesting that the modulation of adaptive immune responses is not their primary function. A structural feature of all TIPs is the presence of FG-GAP repeats, which are present in 2 copies in EmTIP. In mammals, FG-GAP repeat-containing proteins have principally been associated with cell-cell/ECM (extracellular matrix) interactions [46], [47]. Alpha-integrins constitute such a group of proteins, harboring up to 7 copies of the FG-GAP repeat at their N-terminus through which they mediate ligand binding and cell-cell/ECM interactions [48]–[51]. Similar to alpha integrins, a signal sequence and a transmembrane domain are found associated with EmTIP and suggest a membrane-bound/extracellular localization. Furthermore, the localization of EmTIP to the inter-cellular space within parasite larvae indicates a possible role in the cell-cell/ECM interactome as described for FG-GAP repeat-containing proteins [46].

Although EmTIP is predicted to contain a transmembrane domain, we detected considerable amounts of the protein in the E/S fraction of *Echinococcus* primary cell cultures, suggesting that the transmembrane domain only confers transient membrane association or that the protein is post-translationally modified. Indeed, recombinant expression of EmTIP as a full-length protein, including transmembrane domain, did not impair its release from HEK-293T cells as a soluble form when compared to that of a truncated EmTIP variant that was depleted of the C-terminal transmembrane domain (data not shown). Likewise, human TIP also contains a predicted transmembrane domain but is nevertheless released by the cells in significant amounts [36]. Shedding of surface-associated components that mediate cell-cell/ECM interactions (e.g. integrins) is frequently observed during dynamic developmental processes that involve cell migration [52], [53]. In these cases, the membrane-associated factors are usually cleaved off by intra-membrane proteases such as rhomboid proteases or the γ-secretase complex [54]. Interestingly, the dramatic morphological changes that occur early during the *Echinococcus* oncosphere-metacestode transition are highly suggestive of extensive cell migration events [55], [56] and such processes can also be observed in the *in vitro* model of metacestode development from primary cells used in our study [36]. Furthermore, the *E. multilocularis* genome clearly encodes rhomboid/γ-secretase proteases [43] and similar to murine and human TIP, EmTIP contains alpha-helix destabilizing glycine residues [57]–[59] known to facilitate the intra-membrane proteolytic cleavage and release of membrane-tethered proteins [60]. Hence, although further experimentation is clearly needed to shed more light on the function of TIP proteins, we suggest a role of EmTIP in cell-cell/ECM interactions during parasite development which involves cleavage of the protein from the cell surface during cell migratory events that are particularly prominent in the early phase of the oncosphere-metacestode transition.

An antibody directed against the FG-GAP repeat-containing portion of EmTIP, which is usually mediating cell-cell/ECM interactions [48], [61], [62], significantly inhibited *E. multilocularis* stem cell proliferation and, thus, the development of novel metacestode vesicles from stem cells. Since cell-cell/ECM interactions are necessary for stem cell proliferation in various organisms [49], [63]–[65], this suggests that EmTIP antibody-mediated binding could affect *Echinococcus* stem cell interactions with the ECM and other cells. Although the precise mechanism through which the anti-EmTIP antibody inhibits *E. multilocularis* stem cell proliferation remains unknown, it is conceivable that it sequesters soluble EmTIP or, more likely, covers membrane-associated EmTIP, and thus interferes with stem cell/ECM interactions. Irrespective of the mechanism, our data clearly indicate an important role of EmTIP in parasite development during the onset of AE and probably also during metastatic growth of the parasite in long-term infections, which all dependent on parasite stem cell proliferation and differentiation [36], [37], [66], [67]. We therefore suggest EmTIP as a promising target for the development of anti-parasitic therapies that could involve the utilization of specific anti-EmTIP antibody, as reported in the present study, or vaccination strategies. An in-depth assessment of the influence of antibody-mediated EmTIP blockade *in vivo* during *E. multilocularis*-driven infections is currently underway in our laboratory and will shed more light on the potency of EmTIP as a host-protective antigen for AE.

A striking finding of this study is the observation that EmTIP promoted the release of IFN-γ by murine CD4+ T-cells *in vitro*, a cytokine known to promote anti-parasitic responses in *E. multilocularis*-infected hosts [12], [15], [25], [44], [68]. Despite the relatively limited overall sequence homology between EmTIP and human TIP (36% amino acid sequence identity), the parasite molecule obviously retained sufficient structural homology to elicit similar responses in human T-cells as mammalian TIP. This was clearly the case concerning the promotion of IFN-γ release, but not concerning IL-10 release, which could be due to structural differences. Although it still has to be determined which signaling pathways are influenced by either cytokine to result in the release of IFN-γ by T-cells, this effect of EmTIP is unprecedented and provides a first possible explanation for the early Th1 response reported during murine and human AE [3], [4], [9], [29]. Importantly, despite the similarities of the murine and human systems, the promotion of IFN-γ release by EmTIP-stimulated T-cells of human origin needs to be confirmed. Nevertheless, if released *in vivo* as observed in our study *in vitro*, EmTIP could interact with host CD4+ T-cells that are present in the surrounding granuloma [69], [70], potentially leading to IFN-γ release and T-cell polarization towards a potentially parasitocidal Th1 response. In this regard, we consider it unlikely that the parasite releases EmTIP primarily in order to modulate the host immune response. We rather suggest that the primary function of EmTIP lies in supporting early parasite development (e.g. by facilitating cell-cell/ECM interactions), that EmTIP is shed off/released by fulfilling this function and then, as an ortholog of human TIP, is inevitably inducing Th1 polarization when acting on host CD4+ T-cells. In later stages of development, when parasite cell migration events are rather limited and/or the release of EmTIP is prevented by the developing germinal and laminated layers of the metacestode, both the Th2 polarizing activities [15], [17], [71] and the immunosuppressive capabilities [28], [72] of the metacestode products can then dominate the immunomodulatory activities of the parasite.

In conclusion, we herein present a novel *E. multilocularis* factor that could be exploited for the development of novel therapeutic measures against AE. In this regard, experiments to assess the potency of EmTIP blockade as a prophylactic measure for asymptomatic AE seropositive subjects and/or the efficiency of EmTIP as a protective antigen for vaccination against AE are needed and currently underway.

## Supporting Information

Figure S1 **Nucleotide and amino acid sequence of** ***Echinococcus multilocularis*** **T-cell Immunomodulatory Protein (EmTIP).** Splice leader (Lower case) and Poly A tail are boxed. The predicted N-terminal signal sequence is underlined. The FG-GAP repeats are shaded in grey. The potential glycosylation sites (NVT and NFT) are shown in solid boxes. The transmembrane region is delimitated with dashed underlines.
(TIF)

Figure S2 **Assessment of affinity-purified anti-EmTIP antibody.** Western blot of a similar amount of purified Thio-tagged EmTIP on (**A**) parasite-containing liver tissue from infected jirds (**B**), *in vitro* cultivated *E. multilocularis* primary cells (**C**), and *in vitro* cultivated *E. multilocularis* metacestode vesicles (**D**). Pre-immune serum (1), anti-EmTIP immune serum (2) or purified anti-EmTIP antibody (3) were used for detection followed by ECL detection and autoradiography. The positions of the molecular mass markers (in kilodaltons) are shown on the left. The arrow indicates the size of thio-tagged EmTIP (**A**) or natural EmTIP (**B, C, D**) in parasite lysates between 50–60 kDa.
(TIF)

Figure S3 **Recombinant expression and secretion of EmTIP in the HEK-293T cell line.** (**A**) Schematic representation of the Em*tip* sequence cloned into pSecTag2 for recombinant expression. A modified version of Emtip with a c-terminal c-Myc tag (rEmTIP_myc) has been expressed in parallel to investigate secretion of the factor by transfected HEK-293T cells. Western blots of control- (pSecTag2) Em*tip*- (pSecTag2-Em*tip*), and Em*tip*_myc- (pSecTag2-Em*tip_*myc) transfected HEK-293T cell lysates (**B, D**) and secretions (**C, E**). Following immunoprecipitation of E/S products with purified anti-EmTIP (**C**) or anti-c-Myc, 9E10 (**E**), the cell lysates and supernatant immunoprecipitates were probed with purified rabbit anti-EmTIP antibody (**B**, **C**) or anti-c-Myc antibody (**D**, **E**) followed by ECL detection and autoradiography. The arrows indicate the position of recombinant EmTIP on the blots.
(TIF)

## References

[1] Brunetti E, Kern P, Vuitton DA (2010) Expert consensus for the diagnosis and treatment of cystic and alveolar echinococcosis in humans. Acta Trop 114: 1–16. 19931502 10.1016/j.actatropica.2009.11.001

[2] Kern P (2010) Clinical features and treatment of alveolar echinococcosis. Curr Opin Infect Dis 23: 505–512. 20683265 10.1097/QCO.0b013e32833d7516

[3] Gottstein B, Wittwer M, Schild M, Merli M, Leib SL, et al (2010) Hepatic gene expression profile in mice perorally infected with *Echinococcus multilocularis* eggs. PLoS One 5: e9779. 20368974 10.1371/journal.pone.0009779 PMC2848562

[4] Mejri N, Hemphill A, Gottstein B (2010) Triggering and modulation of the host-parasite interplay by *Echinococcus multilocularis*: a review. Parasitology 137: 557–568. 19961650 10.1017/S0031182009991533

[5] Ammann RW, Hirsbrunner R, Cotting J, Steiger U, Jacquier P, et al (1990) Recurrence rate after discontinuation of long-term mebendazole therapy in alveolar echinococcosis (preliminary results). Am J Trop Med Hyg 43: 506–515. 2240375 10.4269/ajtmh.1990.43.506

[6] Ammann RW, Ilitsch N, Marincek B, Freiburghaus AU (1994) Effect of chemotherapy on the larval mass and the long-term course of alveolar echinococcosis. Swiss Echinococcosis Study Group. Hepatology 19: 735–742. 8119701 10.1002/hep.1840190328

[7] WHO Informal Working Group on Echinococcosis (1996) Guidelines for treatment of cystic and alveolar echinococcosis in humans. Bull World Health Organ 74: 231–242. 8789923 PMC2486920

[8] Reuter S, Jensen B, Buttenschoen K, Kratzer W, Kern P (2000) Benzimidazoles in the treatment of alveolar echinococcosis: a comparative study and review of the literature. J Antimicrob Chemother 46: 451–456. 10980173 10.1093/jac/46.3.451

[9] Vuitton DA, Gottstein B (2010) *Echinococcus multilocularis* and its intermediate host: a model of parasite-host interplay. J Biomed Biotechnol 2010: 923193. 20339517 10.1155/2010/923193 PMC2842905

[10] Zhang W, Li J, McManus DP (2003) Concepts in immunology and diagnosis of hydatid disease. Clin Microbiol Rev 16: 18–36. 12525423 10.1128/CMR.16.1.18-36.2003 PMC145297

[11] Godot V, Harraga S, Podoprigora G, Liance M, Bardonnet K, et al (2003) IFN alpha-2a protects mice against a helminth infection of the liver and modulates immune responses. Gastroenterology 124: 1441–1450. 12730883 10.1016/s0016-5085(03)00273-7

[12] Emery I, Liance M, Leclerc C (1997) Secondary *Echinococcus multilocularis* infection in A/J mice: delayed metacestode development is associated with Th1 cytokine production. Parasite Immunol 19: 493–503. 9427996 10.1046/j.1365-3024.1997.d01-162.x

[13] Emery I, Leclerc C, Sengphommachanh K, Vuitton DA, Liance M (1998) In vivo treatment with recombinant IL-12 protects C57BL/6J mice against secondary alveolar echinococcosis. Parasite Immunol 20: 81–91. 9572051 10.1046/j.1365-3024.1998.00131.x

[14] Harraga S, Godot V, Bresson-Hadni S, Pater C, Beurton I, et al (1999) Clinical efficacy of and switch from T helper 2 to T helper 1 cytokine profile after interferon alpha2a monotherapy for human echinococcosis. Clin Infect Dis 29: 205–206. 10433588 10.1086/520157

[15] Godot V, Harraga S, Beurton I, Deschaseaux M, Sarciron E, et al (2000) Resistance/susceptibility to *Echinococcus multilocularis* infection and cytokine profile in humans. I. Comparison of patients with progressive and abortive lesions. Clin Exp Immunol 121: 484–490. 10971515 10.1046/j.1365-2249.2000.01308.x PMC1905721

[16] Emery I, Leclerc C, Houin R, Vuitton DA, Liance M (1997) Lack of H-2 gene influence on mouse susceptibility to secondary alveolar echinococcosis. Int J Parasitol 27: 1433–1436. 9421736 10.1016/s0020-7519(97)00091-x

[17] Dai WJ, Hemphill A, Waldvogel A, Ingold K, Deplazes P, et al (2001) Major carbohydrate antigen of *Echinococcus multilocularis* induces an immunoglobulin G response independent of alphabeta+ CD4+ T cells. Infect Immun 69: 6074–6083. 11553545 10.1128/IAI.69.10.6074-6083.2001 PMC98736

[18] Kilwinski J, Jenne L, Jellen-Ritter A, Radloff P, Flick W, et al (1999) T lymphocyte cytokine profile at a single cell level in alveolar echinococcosis. Cytokine 11: 373–381. 10328877 10.1006/cyto.1998.0432

[19] Wellinghausen N, Gebert P, Kern P (1999) Interleukin (IL)-4, IL-10 and IL-12 profile in serum of patients with alveolar echinococcosis. Acta Trop 73: 165–174. 10465056 10.1016/s0001-706x(99)00027-3

[20] Rau ME, Tanner CE (1975) BCG suppresses growth and metastasis of hydatid infections. Nature 256: 318–319. 1095933 10.1038/256318a0

[21] Reuben JM, Tanner CE, Rau ME (1978) Immunoprophylaxis with BCG of experimental Echinococcus multilocularis infections. Infect Immun 21: 135–139. 711311 10.1128/iai.21.1.135-139.1978 PMC421967

[22] Reuben JM, Tanner CE, Portelance V (1979) Protection of cotton rats against experimental *Echinococcus multilocularis* infections with BCG cell walls. Infect Immun 23: 582–586. 378829 10.1128/iai.23.3.582-586.1979 PMC414205

[23] Luo Y, Yamada H, Evanoff DP, Chen X (2006) Role of Th1-stimulating cytokines in bacillus Calmette-Guerin (BCG)-induced macrophage cytotoxicity against mouse bladder cancer MBT-2 cells. Clin Exp Immunol 146: 181–188. 16968412 10.1111/j.1365-2249.2006.03191.x PMC1809722

[24] Dražilová S, Kinceková J, Bena L, Zachar M, Švajdler M, et al (2012) Regression of alveolar echinococcosis after chronic viral hepatitis C treatment with pegylated interferon alpha 2a. Helminthologia 49: 134–138.

[25] Liance M, Ricard-Blum S, Emery I, Houin R, Vuitton DA (1998) *Echinococcus multilocularis* infection in mice: in vivo treatment with a low dose of IFN-γ decreases metacestode growth and liver fibrogenesis. Parasite 5: 231–237. 9772722 10.1051/parasite/1998053231

[26] Schmid M, Samonigg H, Stoger H, Auer H, Sternthal MH, et al (1995) Use of interferon γ and mebendazole to stop the progression of alveolar hydatid disease: case report. Clin Infect Dis 20: 1543–1546. 7548507 10.1093/clinids/20.6.1543

[27] Mejri N, Muller N, Hemphill A, Gottstein B (2011) Intraperitoneal *Echinococcus multilocularis* infection in mice modulates peritoneal CD4+ and CD8+ regulatory T cell development. Parasitol Int 60: 45–53. 20965274 10.1016/j.parint.2010.10.002

[28] Nono JK, Pletinckx K, Lutz MB, Brehm K (2012) Excretory/secretory-products of *Echinococcus multilocularis* larvae induce apoptosis and tolerogenic properties in dendritic cells *in vitro* . PLoS Negl Trop Dis 6: e1516. 22363826 10.1371/journal.pntd.0001516 PMC3283565

[29] Yang Y, Ellis MK, McManus DP (2012) Immunogenetics of human echinococcosis. Trends Parasitol 28: 447–454. 22951425 10.1016/j.pt.2012.08.001

[30] Zhang W, Ross AG, McManus DP (2008) Mechanisms of immunity in hydatid disease: implications for vaccine development. J Immunol 181: 6679–6685. 18981082 10.4049/jimmunol.181.10.6679

[31] Dvoroznakova E, Porubcova J, Sevcikova Z (2009) Immune response of mice with alveolar echinococcosis to therapy with transfer factor, alone and in combination with albendazole. Parasitol Res 105: 1067–1076. 19548004 10.1007/s00436-009-1520-z

[32] Grzych JM, Pearce E, Cheever A, Caulada ZA, Caspar P, et al (1991) Egg deposition is the major stimulus for the production of Th2 cytokines in murine schistosomiasis mansoni. J Immunol 146: 1322–1327. 1825109

[33] Lawrence RA, Allen JE, Gregory WF, Kopf M, Maizels RM (1995) Infection of IL-4-deficient mice with the parasitic nematode Brugia malayi demonstrates that host resistance is not dependent on a T helper 2-dominated immune response. J Immunol 154: 5995–6001. 7751642

[34] Rodriguez-Sosa M, Saavedra R, Tenorio EP, Rosas LE, Satoskar AR, et al (2004) A STAT4-dependent Th1 response is required for resistance to the helminth parasite *Taenia crassiceps* . Infect Immun 72: 4552–4560. 15271915 10.1128/IAI.72.8.4552-4560.2004 PMC470677

[35] Olson PD, Zarowiecki M, Kiss F, Brehm K (2012) Cestode genomics - progress and prospects for advancing basic and applied aspects of flatworm biology. Parasite Immunol 34: 130–150. 21793855 10.1111/j.1365-3024.2011.01319.x

[36] Spiliotis M, Lechner S, Tappe D, Scheller C, Krohne G, et al (2008) Transient transfection of *Echinococcus multilocularis* primary cells and complete in vitro regeneration of metacestode vesicles. Int J Parasitol 38: 1025–1039. 18086473 10.1016/j.ijpara.2007.11.002

[37] Reuter M, Kreshchenko N (2004) Flatworm asexual multiplication implicates stem cells and regeneration. Canadian Journal of Zoology 82: 334–356.

[38] Fiscella M, Perry JW, Teng B, Bloom M, Zhang C, et al (2003) TIP, a T-cell factor identified using high-throughput screening increases survival in a graft-versus-host disease model. Nat Biotechnol 21: 302–307. 12598909 10.1038/nbt797

[39] Kaczanowski S, Zielenkiewicz P (2003) A TIP on malaria (genomics). Nat Biotechnol 21: 733. 12833085 10.1038/nbt0703-733

[40] Spiliotis M, Brehm K (2009) Axenic in vitro cultivation of *Echinococcus multilocularis* metacestode vesicles and the generation of primary cell cultures. Methods Mol Biol 470: 245–262. 19089387 10.1007/978-1-59745-204-5_17

[41] Brehm K, Jensen K, Frosch M (2000) mRNA trans-splicing in the human parasitic cestode *Echinococcus multilocularis* . J Biol Chem 275: 38311–38318. 10973970 10.1074/jbc.M006091200

[42] Gottstein B, Haag K, Walker M, Matsumoto J, Mejri N, et al (2006) Molecular survival strategies of *Echinococcus multilocularis* in the murine host. Parasitol Int 55 Suppl: S45–S49. 16352460 10.1016/j.parint.2005.11.006

[43] Tsai IJ, Zarowiecki M, Holroyd N, Garciarrubio A, Sanchez-Flores A, et al (2013) The genomes of four tapeworm species reveal adaptations to parasitism. Nature 496: 57–63. 23485966 10.1038/nature12031 PMC3964345

[44] Jenne L, Kilwinski J, Radloff P, Flick W, Kern P (1998) Clinical efficacy of and immunologic alterations caused by interferon γ therapy for alveolar echinococcosis. Clin Infect Dis 26: 492–494. 9502476 10.1086/516316

[45] Manfras BJ, Reuter S, Wendland T, Boehm BO, Kern P (2004) Impeded Th1 CD4 memory T cell generation in chronic-persisting liver infection with *Echinococcus multilocularis* . Int Immunol 16: 43–50. 14688059 10.1093/intimm/dxh005

[46] Steck E, Braun J, Pelttari K, Kadel S, Kalbacher H, et al (2007) Chondrocyte secreted CRTAC1: a glycosylated extracellular matrix molecule of human articular cartilage. Matrix Biol 26: 30–41. 17074475 10.1016/j.matbio.2006.09.006

[47] Velling T, Kusche-Gullberg M, Sejersen T, Gullberg D (1999) cDNA cloning and chromosomal localization of human alpha(11) integrin. A collagen-binding, I domain-containing, beta(1)-associated integrin alpha-chain present in muscle tissues. J Biol Chem 274: 25735–25742. 10464311 10.1074/jbc.274.36.25735

[48] Baneres JL, Roquet F, Martin A, Parello J (2000) A minimized human integrin alpha(5)beta(1) that retains ligand recognition. J Biol Chem 275: 5888–5903. 10681581 10.1074/jbc.275.8.5888

[49] Brizzi MF, Tarone G, Defilippi P (2012) Extracellular matrix, integrins, and growth factors as tailors of the stem cell niche. Curr Opin Cell Biol 24: 645–651. 22898530 10.1016/j.ceb.2012.07.001

[50] Na J, Marsden M, DeSimone DW (2003) Differential regulation of cell adhesive functions by integrin alpha subunit cytoplasmic tails in vivo. J Cell Sci 116: 2333–2343. 12711704 10.1242/jcs.00445

[51] Ramos JW, Whittaker CA, DeSimone DW (1996) Integrin-dependent adhesive activity is spatially controlled by inductive signals at gastrulation. Development 122: 2873–2883. 8787760 10.1242/dev.122.9.2873

[52] Rigort A, Grunewald J, Herzog V, Kirfel G (2004) Release of integrin macroaggregates as a mechanism of rear detachment during keratinocyte migration. Eur J Cell Biol 83: 725–733. 15679117 10.1078/0171-9335-00431

[53] Palecek SP, Huttenlocher A, Horwitz AF, Lauffenburger DA (1998) Physical and biochemical regulation of integrin release during rear detachment of migrating cells. J Cell Sci 111 Pt 7: 929–940. 9490637 10.1242/jcs.111.7.929

[54] Lemberg MK (2011) Intramembrane proteolysis in regulated protein trafficking. Traffic 12: 1109–1118. 21585636 10.1111/j.1600-0854.2011.01219.x

[55] Holcman B, Heath DD, Shaw RJ (1994) Ultrastructure of oncosphere and early stages of metacestode development of *Echinococcus granulosus* . Int J Parasitol 24: 623–635. 7928063 10.1016/0020-7519(94)90114-7

[56] Harris A, Heath DD, Lawrence SB, Shaw RJ (1989) Echinococcus granulosus: ultrastructure of epithelial changes during the first 8 days of metacestode development *in vitro* . Int J Parasitol 19: 621–629. 2807718 10.1016/0020-7519(89)90040-4

[57] Chakrabartty A, Schellman JA, Baldwin RL (1991) Large differences in the helix propensities of alanine and glycine. Nature 351: 586–588. 2046766 10.1038/351586a0

[58] Lopez-Llano J, Campos LA, Sancho J (2006) Alpha-helix stabilization by alanine relative to glycine: roles of polar and apolar solvent exposures and of backbone entropy. Proteins 64: 769–778. 16755589 10.1002/prot.21041

[59] Pace CN, Scholtz JM (1998) A helix propensity scale based on experimental studies of peptides and proteins. Biophys J 75: 422–427. 9649402 10.1016/s0006-3495(98)77529-0 PMC1299714

[60] Moin SM, Urban S (2012) Membrane immersion allows rhomboid proteases to achieve specificity by reading transmembrane segment dynamics. Elife 1: e00173. 23150798 10.7554/eLife.00173 PMC3494066

[61] Springer TA (1997) Folding of the N-terminal, ligand-binding region of integrin alpha-subunits into a beta-propeller domain. Proc Natl Acad Sci U S A 94: 65–72. 8990162 10.1073/pnas.94.1.65 PMC19237

[62] Kamata T, Tieu KK, Irie A, Springer TA, Takada Y (2001) Amino acid residues in the alpha IIb subunit that are critical for ligand binding to integrin alpha IIbbeta 3 are clustered in the beta-propeller model. J Biol Chem 276: 44275–44283. 11557768 10.1074/jbc.M107021200

[63] Ellis SJ, Tanentzapf G (2010) Integrin-mediated adhesion and stem-cell-niche interactions. Cell Tissue Res 339: 121–130. 19588168 10.1007/s00441-009-0828-4

[64] Marthiens V, Kazanis I, Moss L, Long K, Ffrench-Constant C (2010) Adhesion molecules in the stem cell niche–more than just staying in shape? J Cell Sci 123: 1613–1622. 20445012 10.1242/jcs.054312 PMC2864709

[65] Raymond K, Deugnier MA, Faraldo MM, Glukhova MA (2009) Adhesion within the stem cell niches. Curr Opin Cell Biol 21: 623–629. 19535237 10.1016/j.ceb.2009.05.004

[66] Matsuhisa T (1996) [The mechanism of distant metastases of alveolar hydatid disease]. Hokkaido Igaku Zasshi 71: 369–376. 8752531

[67] Mehlhorn H, Eckert J, Thompson RC (1983) Proliferation and metastases formation of larval *Echinococcus multilocularis*. II. Ultrastructural investigations. Z Parasitenkd 69: 749–763. 6659652 10.1007/BF00927424

[68] Emery I, Liance M, Deriaud E, Vuitton DA, Houin R, et al (1996) Characterization of T-cell immune responses of *Echinococcus multilocularis*-infected C57BL/6J mice. Parasite Immunol 18: 463–472. 9226682 10.1111/j.1365-3024.1996.tb01030.x

[69] Vuitton DA, Bresson-Hadni S, Laroche L, Kaiserlian D, Guerret-Stocker S, et al (1989) Cellular immune response in *Echinococcus multilocularis* infection in humans. II. Natural killer cell activity and cell subpopulations in the blood and in the periparasitic granuloma of patients with alveolar echinococcosis. Clin Exp Immunol 78: 67–74. 2805425 PMC1534601

[70] Wang J, Zhang C, Wei X, Blagosklonov O, Lv G, et al (2013) TGF-beta and TGF-beta/Smad signaling in the interactions between *Echinococcus multilocularis* and its hosts. PLoS One 8: e55379. 23405141 10.1371/journal.pone.0055379 PMC3566151

[71] Issaadi N, Fraize M, Azzouz S, Petavy AF, Sarciron ME (2006) *Echinococcus multilocularis*: immunity response to purified alkaline phosphatase in BALB/c mice. Parasitol Res 98: 218–226. 16333665 10.1007/s00436-005-0041-7

[72] Hubner MP, Manfras BJ, Margos MC, Eiffler D, Hoffmann WH, et al (2006) *Echinococcus multilocularis* metacestodes modulate cellular cytokine and chemokine release by peripheral blood mononuclear cells in alveolar echinococcosis patients. Clin Exp Immunol 145: 243–251. 16879243 10.1111/j.1365-2249.2006.03142.x PMC1809686

