# Supplementary figures and images for: EmTIP, a T-Cell Immunomodulatory Protein Secreted by the Tapeworm Echinococcus multilocularis Is Important for Early Metacestode Development
Source: PLoS Negl Trop Dis. 2014 Jan 2;8(1):e2632. doi: 10.1371/journal.pntd.0002632 (PMC3879249; doi:10.1371/journal.pntd.0002632)

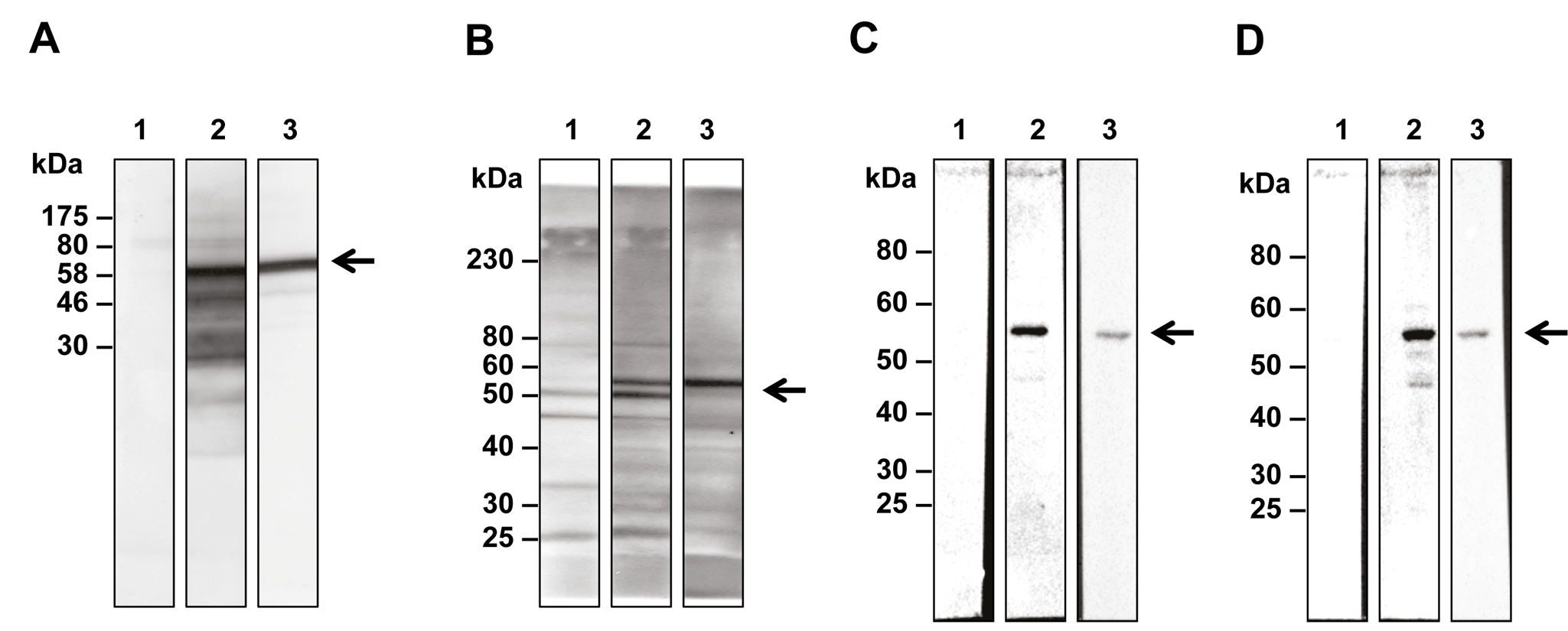

Supplement: Figure S2 — Assessment of affinity-purified anti-EmTIP antibody. Western blot of a similar amount of purified Thio-tagged EmTIP on (A) parasite-containing liver tissue from infected jirds (B), in vitro cultivated E. multilocularis primary cells (C), and in vitro cultivated E. multilocularis metacestode vesicles (D). Pre-immune serum (1), anti-EmTIP immune serum (2) or purified anti-EmTIP antibody (3) were used for detection followed by ECL detection and autoradiography. The positions of the molecular mass markers (in kilodaltons) are shown on the left. The arrow indicates the size of thio-tagged EmTIP (A) or natural EmTIP (B, C, D) in parasite lysates between 50–60 kDa. (TIF) [file pntd.0002632.s002.tif]

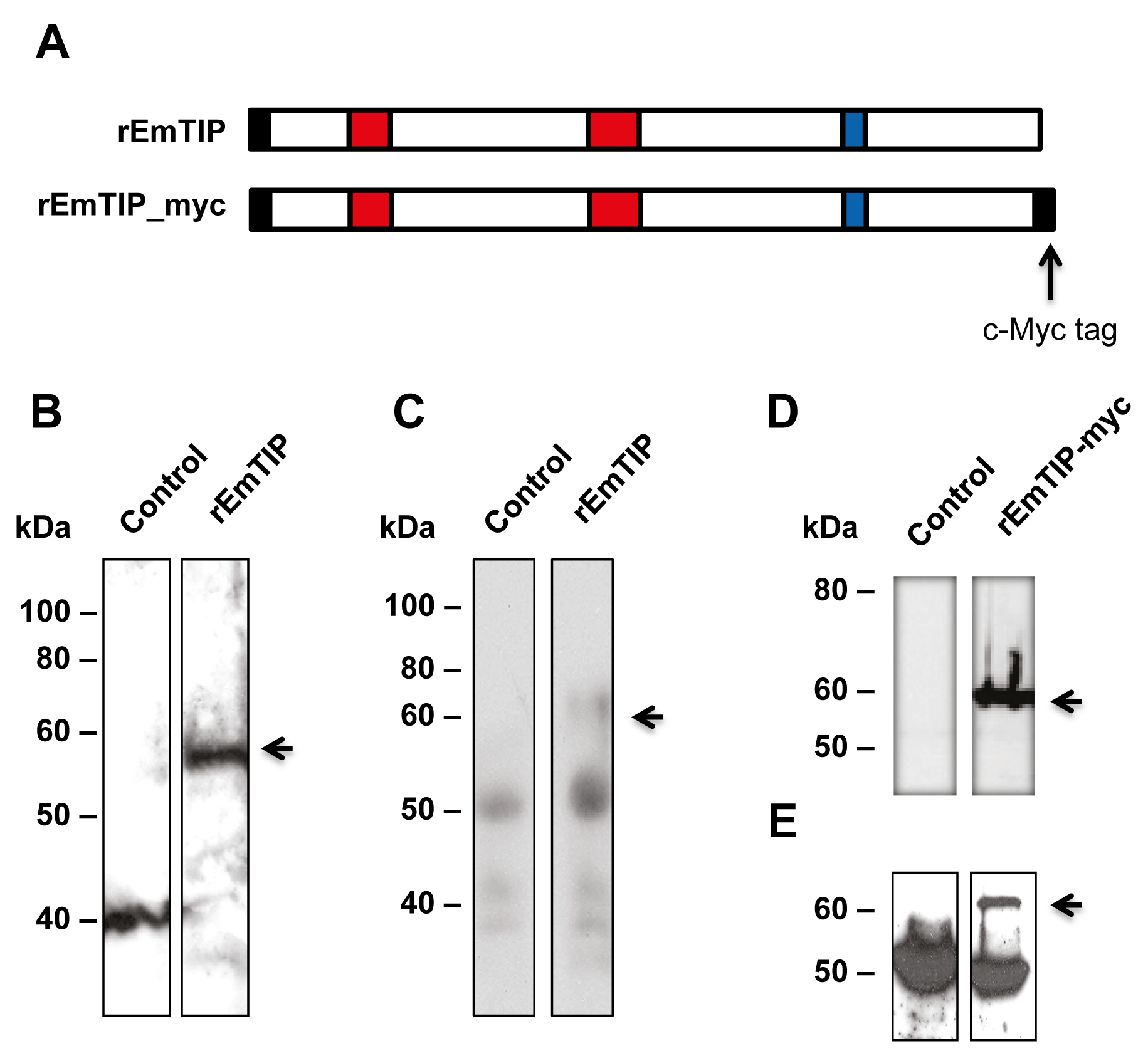

Supplement: Figure S3 — Recombinant expression and secretion of EmTIP in the HEK-293T cell line. (A) Schematic representation of the Emtip sequence cloned into pSecTag2 for recombinant expression. A modified version of Emtip with a c-terminal c-Myc tag (rEmTIP_myc) has been expressed in parallel to investigate secretion of the factor by transfected HEK-293T cells. Western blots of control- (pSecTag2) Emtip- (pSecTag2-Emtip), and Emtip_myc- (pSecTag2-Emtip_myc) transfected HEK-293T cell lysates (B, D) and secretions (C, E). Following immunoprecipitation of E/S products with purified anti-EmTIP (C) or anti-c-Myc, 9E10 (E), the cell lysates and supernatant immunoprecipitates were probed with purified rabbit anti-EmTIP antibody (B, C) or anti-c-Myc antibody (D, E) followed by ECL detection and autoradiography. The arrows indicate the position of recombinant EmTIP on the blots. (TIF) [file pntd.0002632.s003.tif]
